# The epidemiology and clinical characteristics of carbapenem-resistant *Enterobacteriaceae* (CRE) isolates from children in eastern China

**DOI:** 10.3389/fmicb.2026.1734516

**Published:** 2026-05-28

**Authors:** Yihan Zheng, Peng Mo, Dan Li, Lin Zhang, Yuqing Wang, Canhong Zhu, Yongdong Yan, Chuangli Hao, Xuejun Shao, Yang Li, Zhengrong Chen

**Affiliations:** 1 Department of Respiratory Medicine, Children’s Hospital of Soochow University, Suzhou, Jiangsu, China; 2 Department of Clinical Laboratory, The Second Affiliated Hospital of Soochow University, Suzhou, Jiangsu, China; 3 Department of Clinical Laboratory, Children’s Hospital of Soochow University, Suzhou, Jiangsu, China

**Keywords:** antimicrobial resistance genes, carbapenem-resistant *Enterobacteriaceae*, children, hypervirulence, hypervirulence associated genes, multidrug resistance

## Abstract

**Introduction:**

Carbapenem-resistant *Enterobacteriaceae* (CRE) is the most common clinical pathogens. Investigating the antimicrobial resistance, hypervirulence and clinical characteristics of CRE isolated from children is helpful to guide for anti-infection treatments.

**Methods:**

Nonduplicated CRE clinical strains were isolated and mass spectrometry was applied to identify clinical isolated strains. VITEK 2 Compact system and Kirby-Bauer method were used to analyze the antimicrobial susceptibility. Besides, the drug resistance and hypervirulence associated genes were detected by polymerase chain reaction (PCR) and sequencing.

**Results:**

A total of 281 non-duplicated CRE strains were identified in this study. *Klebsiella pneumoniae* (55.87%), *Escherichia coli* (36.65%) and *Klebsiella aerogenes* (3.20%) were the top 3 CRE strains. These strains showed high resistance to most of antimicrobial agents and carried carbapenemase genes, including *bla*_OXA-232_ (38.08%), *bla*_KPC-2_ (20.28%), *bla*_OXA-1_ (17.08%), *bla*_NDM-5_ (10.32%) and *bla*_NDM-1_ (8.90%). In addition, *bla*_TEM-1_ (98.93%), *bla*_CTX-M-14_ (88.61%) and *bla*_SHV-11_ (87.54%) were the prevalent extended-spectrum β-lactamase (ESBL) genes in these strains, while the detection rate of AmpC cephalosporinase genes were not high. Besides, 45 (28.66%) carbapenem-resistant *Klebsiella pneumoniae* (CRKP) strains carried hypervirulence associated genes *iucA* (24.84%), *_p_rmpA* (17.83%), *peg-344* (17.20%), and *_p_rmpA2* (9.55%). Among them, 22 (14.01%) CRKP strains were also identified as carbapenem-resistant and hypervirulent *Klebsiella pneumoniae* (CR-HVKP). What’s worse, the patients infected with CR-HVKP had a worse prognosis overall.

**Conclusion:**

This study revealed the drug resistance, hypervirulence and epidemiology of CRE strains in pediatric patients in Suzhou of eastern China. Unfortunately, CR-HVKP strains with more several infection were also identified, which should be of great concern to clinicians.

## Introduction

Carbapenem-resistant *Enterobacteriaceae* (CRE) is the most common gram-negative bacteria that will cause multiple parts of infections, including respiratory tract, urinary tract infection, digestive tract, skin, blood and cerebrospinal fluid (Perez and Bonomo, 2019; Nordmann et al., 2012). It has been widely spread and become one of the serious challenges in public health globally (Logan and Weinstein, 2017). The infection caused by CRE often has high drug resistance and mortality, with worse prognosis in children (Logan et al., 2015). However, the isolation rate of CRE in children is still increasing and is higher than that in adults in China (Zhang et al., 2018; Hu et al., 2018).

The misuse and overuse of antibiotics resulted in the development of drug resistance and the emergence of CRE in recent years (Miao et al., 2019). The drug-resistance mechanism of CRE is mainly due to the production of carbapenemase, mainly including *Klebsiella pneumoniae* carbapenemase (KPC), Verona integron-encoded metallo-β-lactamase (VIM), New Delhi metallo-beta-lactamase (NDM), Imipenemase (IMP) and Oxacillinase (OXA), and they are able to hydrolyze carbapenem antibiotics, like imipenem, meropenem and ertapenem (Miao et al., 2019; Nordmann and Poirel, 2014). In addition, CRE strains also carry genes for resistance to other antimicrobial agents, like extended-spectrum β-lactamase (ESBL) and AmpC cephalosporinase genes, showing wide range of resistance to antimicrobial drugs (Logan and Weinstein, 2017; Miao et al., 2019). Therefore, the extensively drug-resistance or even Pan-drug resistance of CRE make it difficult for clinical anti-infection treatment.

In recent years, hypervirulent *Enterobacteriaceae* has been reported in many countries and has also become a public health concern globally, like hypervirulent *Klebsiella pneumoniae* (HVKP) and hypervirulent *Escherichia coli* (HVEC) (Russo and Marr, 2019; Divya and Hatha, 2019). The hypervirulence is usually associated with capsule, siderophore and other virulence factors. These hypervirulent strains have the ability to cause more severe infections than non-hypervirulent strains and threat life (Walker and Miller, 2020; Russo et al., 2014). The definition of hypervirulence is problematic without consensus. Previous studies defined hypervirulence as the presence of hypervirulence-associated genes combined with a positive string test. This approach may underestimate hypervirulent strains lacking hypermucoviscosity and misclassify strains without functional validation. Therefore, this definition represents a surrogate marker of hypervirulence rather than definitive proof (Yang et al., 2025).

What’s worse, the overlapping of carbapenem-resistance and hypervirulence were also reported in the same bacteria strain, which had a worse prognosis and should be of concern (Yao et al., 2018; Karlsson et al., 2019; Gu et al., 2018). However, there have been limited studies about the overlapping of carbapenem-resistance and hypervirulence in strains isolated from pediatric patients. Therefore, we investigated the characteristics of antibiotic resistance and hypervirulence of CRE isolates from children at a children’s medical center in eastern China in the present study.

## Materials and methods

### Study site

This study was conducted in Children’s Hospital of Soochow University (CHSU), which is the children’s medical center in eastern China and the only provincial tertiary children’s hospital in Jiangsu Province, China. CHSU has 1,500 beds, and serves >77,000 inpatients and >2.5 million outpatients in 2021. This study had no impact on patients and was approved by the Ethics Committee of CHSU (No. 2020CS099).

### CRE strains

Nonduplicated CRE clinical isolates were collected at CHSU from January 1, 2016, to December 31, 2020. In this study, the strains were identified by matrix-assisted laser desorption ionization time-of-flight mass spectrometry (MALDI-TOF MS, Bruker, Mannheim, Germany), and carbapenem resistance was defined as resistance to meropenem (MIC ≥ 4 μg/mL), imipenem (MIC ≥ 4 μg/mL) or ertapenem (MIC ≥ 2 μg/mL) based on 2021 Clinical and Laboratory Standards Institute (CLSI) guidelines (Li et al., 2021b).

### Antimicrobial susceptibility test

The antimicrobial susceptibility of CRE clinical isolates was analyzed by the Vitek 2 Compact system (bioMérieux, Marcy-l’Etoile, France) and Kirby-Bauer (K-B) method according to the manufacturer’s instructions. A total of 20 antibiotics belonging to 11 classes of antimicrobials were tested. In addition, *Escherichia coli* (ATCC 25922) and *Pseudomonas aeruginosa* (ATCC 27853) were used for quality control (Li et al., 2021a).

### Detection of antimicrobial resistance genes

Three kinds of carbapenem-resistant genes, including carbapenemase genes (*bla*_KPC_, *bla*_OXA_, *bla*_NDM_, *bla*_VIM_, *bla*_IMP_, *bla*_AIM_, *bla*_GIM_, *bla*_SIM_, *bla*_SPM_, *bla*_BIC_ and *bla*_DIM_), extended-spectrum *β*-lactamase (ESBL) genes (*bla*_TEM_, *bla*_SHV_, *bla*_CTX-M_, *bla*_VEB_ and *bla*_PER_) and AmpC cephalosporinase genes (*bla*_CMY_, *bla*_ACT_, *bla*_ACC_, *bla*_DHA_, *bla*_FOX_, *bla*_MOX_, *bla*_CIT_ and *bla*_EBC_), were amplified by PCR and their PCR products were analyzed by agarose gel electrophoresis. Positive products were sequenced, and then aligned by using the Basic Local Alignment Search Tool (BLAST) in GenBank. Primers used for detecting the above antimicrobial resistance genes have been reported previously (Li et al., 2021b; Wang et al., 2020).

### Identification of hypervirulent strains

Eight hypervirulence-associated genes (*iucA*, *_p_rmpA*, *_p_rmpA2*, *_c_rmpA, peg-344*, *terB*, *iroB* and *irp2*) of *Klebsiella pneumoniae* and 3 hypervirulence-associated genes (*Stx1*, *Stx2*, *eae*) of *Escherichia coli*, which have been reported previously, were screened by PCR as described above (Divya and Hatha, 2019; Li et al., 2021b; Russo et al., 2018; Szczerba-Turek et al., 2020). In addition, the strains with hypervirulence-associated genes were further detected by string test as described previously to determine the hypermucoviscous phenotype (Zhou et al., 2021). The strains with a positive string test (hypermucoviscosity) and co-harboring the hypervirulence-associated genes at the same time were defined as hypervirulent strains (Russo et al., 2018; Yu et al., 2018).

### Data analysis

The antimicrobial susceptibility data of CRE clinical isolates were analyzed with WHONET software (WHO Collaborating Centre for Surveillance of Antimicrobial Resistance, Boston, MA, United States). Chi-squared test was performed to analyze the data and *p* < 0.05 was considered statistically significant.

## Results

### Epidemiology and clinical characteristics of CRE infection

**Figure 1:**
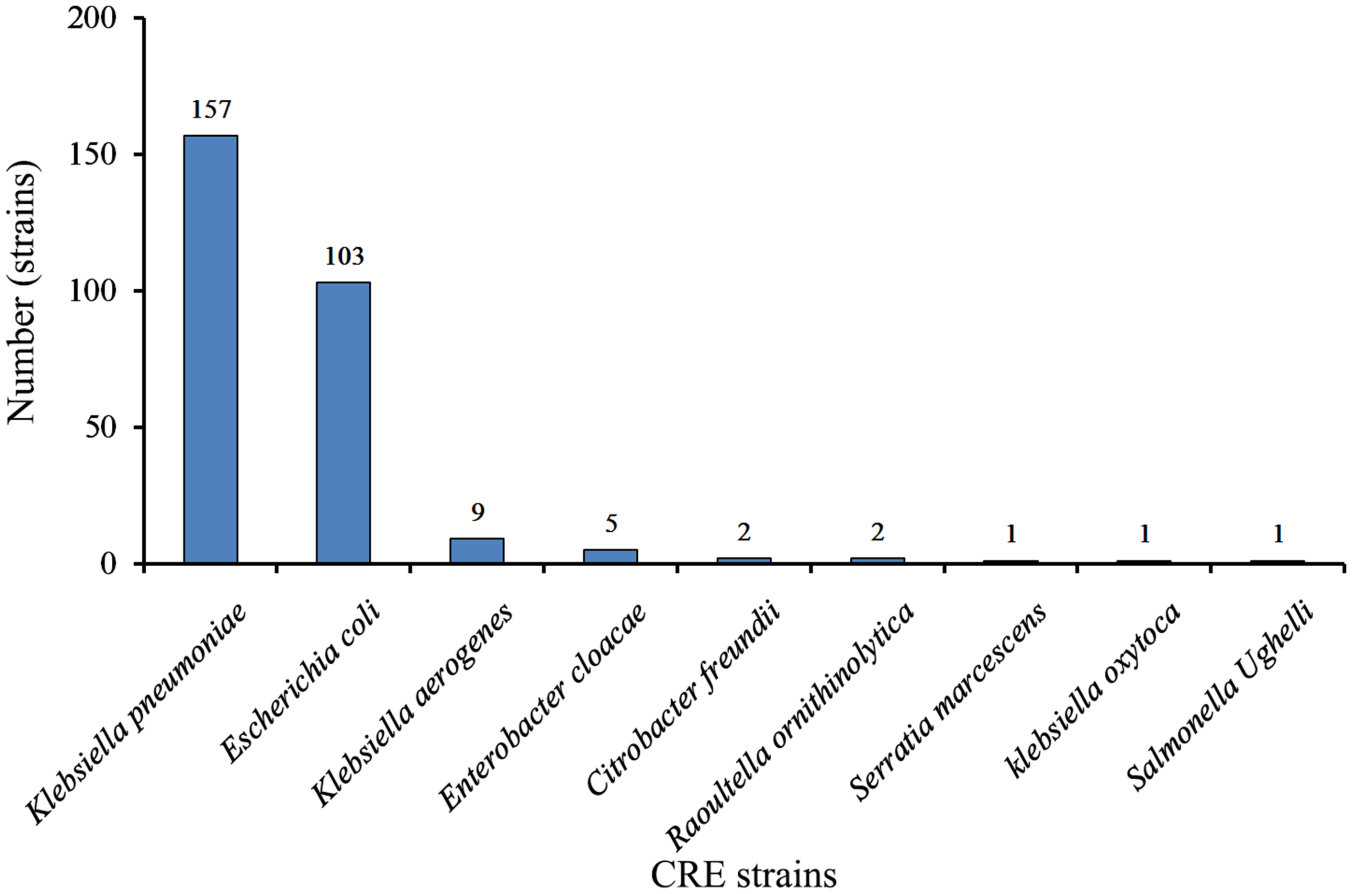
The number of CRE strains isolated from pediatric patients at Children’s Hospital of Soochow University from 2016 to 2020.

**Figure 2:**
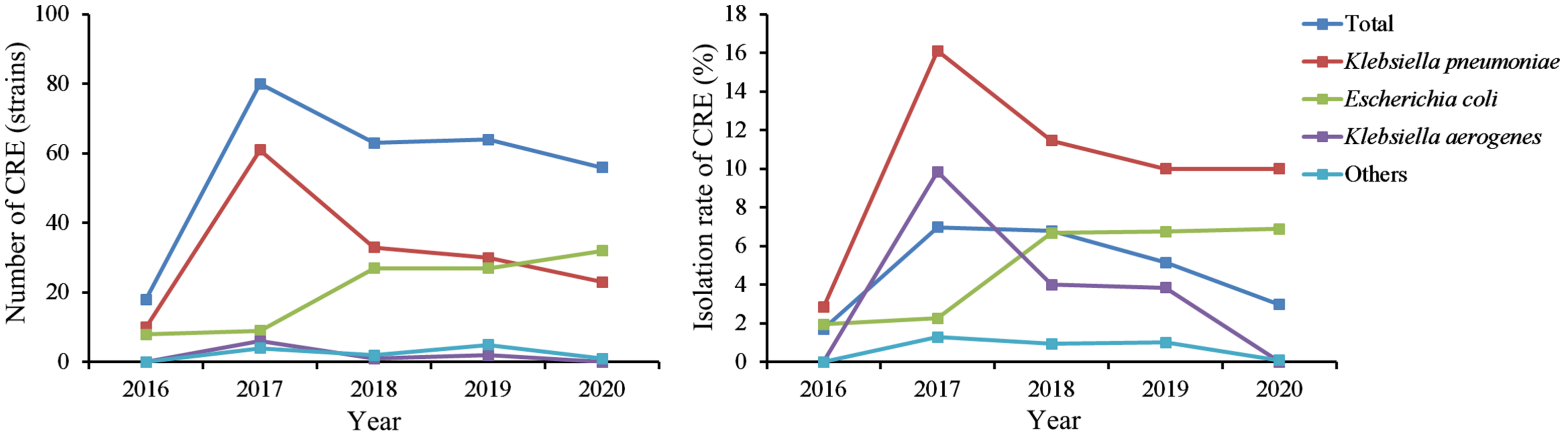
The annual trends of CRE number and isolation rate from 2016 to 2020 in this study.

A total of 281 non-duplicated CRE isolated strains were collected and identified in this 5-year study, consisting of 157 *Klebsiella pneumoniae*, 103 *Escherichia coli*, 9 *Klebsiella aerogenes*, 5 *Enterobacter cloacae*, 2 *Citrobacter freundii*, 2 *Raoultella ornithinolytica*, 1 *Serratia marcescens*, 1 *klebsiella oxytoca* and 1 *Salmonella Ughelli* (Figure 1). The CRE strain number and isolation rate both increased from 2016 to 2017 due to the remarkable increase of carbapenem-resistant *Klebsiella pneumoniae* and *Klebsiella aerogenes* in 2017. However, the CRE isolation rate decreased annually during 2018–2020. In addition, the number and isolation rate of carbapenem-resistant *Escherichia coli* increased slightly in 2018–2020 (Figure 2).

The clinical characteristics of pediatric patients in this study are summarized in Table 1. Among the 281 pediatric patients, 134 (47.69%) were male and the median age was 1 month (range from 1 h to 16 years old). The majority of children (90.75%) were aged <5 years at the time of diagnosis. These CRE strains were isolated from urine (40.57%), sputum (40.21%), blood (11.74%), alveolar lavage fluid (2.14%), Skin pus (1.78%) and other samples (3.56%). Most of the pediatric patients were in the department of neonatology (34.52%), neonatal intensive care unit (NICU, 12.81%), hematology (11.03%), pediatric intensive care unit (PICU, 10.32%) and nephrology (6.76%). In addition, the diagnose diseases of these patients were pneumonia (61.56%), urinary tract infection (33.81%), icterus (21.35%) and septicemia (21.35%). However, a part of children had underlying health condition, like cardiac diseases (15.30%), leukemia (11.39%), urinary disease (4.98), epilepsy (2.14%) and respiratory disease (1.78%). After the anti-infective treatment by antibiotics, the condition of 235 pediatric patients (83.63%) improved and the median time of hospitalization was 27 days (range from 4 to 210 days).

**Table 1:** Clinical characteristics and epidemiology of CRE infection at Children’s Hospital of Soochow University from 2016 to 2020.

	Total	*Klebsiella pneumoniae*	*Escherichia coli*	*Klebsiella aerogenes*	Others
	*n* = 281	*n* = 157	*n* = 103	*n* = 9	*n* = 12
Gender, *n* (%)					
Male	134 (47.69)	85 (54.14)	38 (36.89)	5 (55.56)	6 (50.00)
Female	147 (52.31)	72 (45.86)	65 (63.11)	4 (44.44)	6 (50.00)
Age					
Median	1 m	1 m	1 m	1 m	2 y
Range	1 h - 16 y	1 h - 16 y	2 h - 13 y	2 h - 8 m	7 d - 13 y
Age, *n* (%)					
<5 y	255 (90.75)	145 (92.36)	91 (88.35)	9 (100.00)	10 (83.33)
<1 m	126 (44.84)	74 (47.13)	45 (43.69)	4 (44.44)	3 (25.00)
1–6 m	81 (28.83)	47 (29.94)	29 (28.16)	4 (44.44)	1 (8.33)
7–12 m	17 (6.05)	10 (6.37)	5 (4.85)	1 (11.11)	1 (8.33)
13–59 m	31 (11.03)	14 (8.92)	12 (11.65)	0 (0)	5 (41.67)
5–18 y	26 (9.25)	12 (7.64)	12 (11.65)	0 (0)	2 (16.67)
Specimen					
Urine	114 (40.57)	52 (33.12)	55 (53.40)	1 (11.11)	6 (50.00)
Sputum	113 (40.21)	75 (47.77)	28 (27.18)	7 (77.78)	3 (25.00)
Blood	33 (11.74)	15 (9.55)	16 (15.53)	1 (11.11)	1 (8.33)
Alveolar lavage fluid	6 (2.14)	5 (3.18)	1 (0.97)	0 (0)	0 (0)
Skin pus	5 (1.78)	3 (1.91)	2 (1.94)	0 (0)	0 (0)
Others	10 (3.56)	7 (4.46)	1 (0.97)	0 (0)	2 (16.67)
Wards, *n* (%)					
Neonatology	97 (34.52)	46 (29.30)	42 (40.78)	4 (44.44)	5 (41.67)
NICU	36 (12.81)	28 (17.83)	7 (6.80)	1 (11.11)	0 (0)
Hematology	31 (11.03)	17 (10.83)	13 (12.62)	0 (0)	1 (8.33)
PICU	29 (10.32)	18 (11.46)	9 (8.74)	1 (11.11)	1 (8.33)
Nephrology	19 (6.76)	9 (5.73)	9 (8.74)	0 (0)	1 (8.33)
Others	69 (24.56)	39 (24.84)	23 (22.33)	3 (33.33)	4 (33.33)
Diseases					
Pneumonia	173 (61.56)	104 (66.24)	60 (58.25)	6 (66.67)	3 (25.00)
Urinary tract infection	95 (33.81)	39 (24.84)	51 (49.51)	1 (11.11)	4 (33.33)
Icterus	60 (21.35)	42 (26.75)	15 (14.56)	2 (22.22)	1 (8.33)
Septicemia	60 (21.35)	32 (20.38)	25 (24.27)	2 (22.22)	1 (8.33)
Underlying condition					
Cardiac diseases	43 (15.30)	21 (13.38)	18 (17.48)	3 (33.33)	1 (8.33)
Leukemia	32 (11.39)	19 (12.10)	12 (11.65)	0 (0)	1 (8.33)
Urinary disease	14 (4.98)	7 (4.46)	6 (5.83)	0 (0)	1 (8.33)
Epilepsy	6 (2.14)	5 (3.18)	1 (0.97)	0 (0)	0 (0)
Respiratory disease	5 (1.78)	2 (1.27)	3 (2.91)	0 (0)	0 (0)
Antibiotics use					
Latamoxef	93 (33.10)	51 (32.48)	36 (34.95)	2 (22.22)	4 (33.33)
Cefoperazone/sulbactam	70 (24.91)	50 (31.85)	18 (17.48)	1 (11.11)	1 (8.33)
Meropenem	44 (15.66)	22 (14.01)	18 (17.48)	3 (33.33)	1 (8.33)
Imipenem	35 (12.46)	25 (15.92)	8 (7.77)	0 (0)	2 (16.67)
Piperacillin/tazobactam	22 (7.83)	15 (9.55)	6 (5.83)	1 (11.11)	0 (0)
Others	109 (38.79)	62 (39.49)	39 (37.86)	3 (33.33)	5 (41.67)
Hospitalization					
Mean	27 d	28 d	22 d	33 d	17 d
Range	4 d–210 d	4 d–116 d	4 d–210 d	8 d–71 d	18 d–28 d
Antimicrobial therapy outcome					
Effective	235 (83.63)	120 (76.43)	98 (95.15)	7 (77.78)	10 (83.33)

*d, days; m, months; y, years.

### Antimicrobial susceptibility

All CRE isolates were resistant to meropenem and imipenem, and exhibited high resistance rates to cefazolin, cefuroxime, ceftriaxone, ceftazidime, cefepime, ceftizoxime, cefoperazone/sulbactam, cefoxitin, cefotetan, aztreonam, ampicillin/sulbactam, piperacillin/tazobactam, piperacillin and sulfamethoxazole trimethoprim (Table 2). The most active antibiotics against all isolates were gentamicin (80.43% susceptible), tobramycin (70.46% susceptible), levofloxacin (64.06% susceptible) and ciprofloxacin (50.89% susceptible). In addition, *Klebsiella pneumoniae* showed high resistance to antimicrobial agents except gentamicin, tobramycin and levofloxacin, while *Escherichia coli* exhibited high resistance to all antimicrobial drugs in this study. However, *Klebsiella aerogenes* showed more sensitivity to drugs, including gentamicin, tobramycin, cefazolin, cefuroxime, cefoxitin, ciprofloxacin, levofloxacin, ampicillin/sulbactam and sulfamethoxazoletrimethoprim (Table 2).

**Table 2:** The antimicrobial resistance of CRE strains in this study.

Antibiotics	Total	*Klebsiella pneumoniae*	*Escherichia coli*	*Klebsiella aerogenes*	Others
	*n* = 281	(*n* = 157)	(*n* = 103)	(*n* = 9)	(*n* = 12)
Aminoglycosides					
Gentamicin	55 (19.57)	17 (10.83)	33 (32.04)	1 (11.11)	4 (33.33)
Tobramycin	83 (29.54)	17 (10.83)	64 (62.14)	0 (0)	2 (16.67)
Carbapenems					
Meropenem	281 (100.00)	157 (100.00)	103 (100.00)	9 (100.00)	12 (100.00)
Imipenem	281 (100.00)	157 (100.00)	103 (100.00)	9 (100.00)	12 (100.00)
Non-extended spectrum cephalosporins; 1st and 2nd generation cephalosporins					
Cefazolin	263 (93.59)	156 (99.36)	100 (97.09)	2 (22.22)	5 (41.67)
Cefuroxime	262 (93.24)	155 (98.73)	101 (98.06)	2 (22.22)	4 (33.33)
Extended-spectrum cephalosporins; 3rd and 4th generation cephalosporins					
Ceftriaxone	272 (96.80)	152 (96.82)	100 (97.09)	9 (100.00)	11 (91.67)
Ceftazidime	260 (92.53)	146 (92.99)	97 (94.17)	7 (77.78)	10 (83.33)
Cefepime	227 (80.78)	124 (78.98)	88 (85.44)	9 (100.00)	6 (50.00)
Ceftizoxime	255 (90.75)	144 (91.72)	96 (93.20)	5 (55.56)	10 (83.33)
Cephalosporins + β-lactamase inhibitors					
Cefoperazone/sulbactam	256 (91.10)	146 (92.99)	94 (91.26)	6 (66.67)	10 (83.33)
Cephamycins					
Cefoxitin	252 (89.68)	149 (94.90)	96 (93.20)	2 (22.22)	5 (41.67)
Cefotetan	244 (86.83)	137 (87.26)	87 (84.47)	8 (88.89)	12 (100.00)
Fluoroquinolones					
Ciprofloxacin	138 (49.11)	49 (31.21)	84 (81.55)	1 (11.11)	4 (33.33)
Levofloxacin	101 (35.94)	17 (10.83)	83 (80.58)	0 (0)	1 (8.33)
Monobactams					
Aztreonam	182 (64.77)	83 (52.87)	85 (82.52)	8 (88.89)	6 (50.00)
Penicillins + β-lactamase inhibitors					
Ampicillin/sulbactam	258 (91.81)	154 (98.09)	100 (97.09)	0 (0)	4 (33.33)
Piperacillin/tazobactam	185 (65.84)	99 (63.06)	79 (76.70)	4 (44.44)	3 (25.00)
Penicillins					
Piperacillin	147 (52.31)	99 (63.06)	40 (38.83)	5 (55.56)	3 (25.00)
Sulfonamides					
Sulfamethoxazole trimethoprim	159 (56.58)	64 (40.76)	89 (86.41)	0 (0)	6 (50.00)

### Distribution of carbapenem-resistant genes

The main carbapenem-resistant genes in *Enterobacteriaceae* are carbapenemase, ESBL and AmpC cephalosporinase genes (Miao et al., 2019; Wang et al., 2020). In this study, these CRE isolates carried carbapenemase genes, including *bla*_OXA-232_ (38.08%), *bla*_KPC-2_ (20.28%), *bla*_OXA-1_ (17.08%), *bla*_NDM-5_ (10.32%) and *bla*_NDM-1_ (8.90%). In addition, *bla*_TEM-1_ (98.93%), *bla*_CTX-M-14_ (88.61%) and *bla*_SHV-11_ (87.54%) were the most prevalent ESBL genes in these CRE strains. However, the percentage of AmpC cephalosporinase genes was not as high as carbapenemase and ESBL genes, only *bla*_DHA-1_ (26.33%) being detected (Table 3). Besides, carbapenem-resistant genes were all detected in *Klebsiella pneumoniae*, *Escherichia coli*, *Klebsiella aerogenes* and *Enterobacter cloacae* (Table 3). These results explained the high drug resistance rate of these CRE isolates.

**Table 3:** The drug resistance genes of CRE strains in this study.

	Total	*Klebsiella pneumoniae*	*Escherichia coli*	*Klebsiella aerogenes*	Others
	*n* = 281	(*n* = 157)	(*n* = 103)	(*n* = 9)	(*n* = 12)
Carbapenemase genes, *n* (%)					
*bla*_NDM-1_	25 (8.90)	18 (11.46)	4 (3.88)	2 (22.22)	1 (8.33)
*bla*_OXA-1_	48 (17.08)	25 (15.92)	19 (18.45)	4 (44.44)	0 (0)
*bla*_KPC-2_	57 (20.28)	39 (24.84)	15 (14.56)	1 (11.11)	2 (16.67)
*bla*_NDM-5_	29 (10.32)	20 (12.74)	9 (8.74)	0 (0)	0 (0)
*bla*_OXA-232_	107 (38.08)	53 (33.76)	54 (52.43)	0 (0)	0 (0)
*bla*_VIM_ *, bla*_IMP_ *, bla*_AIM_ *, bla*_GIM_ *, bla*_SIM_ *, bla*_SPM_ *, bla*_BIC_ *, bla*_DIM_	0 (0)	0 (0)	0 (0)	0 (0)	0 (0)
ESBL genes, *n* (%)					
*bla*_TEM-1_	278 (98.93)	157 (100.00)	101 (98.06)	9 (100.00)	11 (91.67)
*bla*_CTX-M-14_	249 (88.61)	133 (84.71)	96 (93.20)	8 (88.89)	12 (100.00)
*bla*_SHV-11_	246 (87.54)	151 (96.17)	79 (76.70)	9 (100.00)	7 (58.33)
*bla*_VEB,_ *bla*_PER_	0 (0)	0 (0)	0 (0)	0 (0)	0 (0)
AmpC enzyme genes, *n* (%)					
*bla*_DHA-1_	74 (26.33)	38 (24.20)	32 (31.07)	1 (11.11)	3 (25.00)
*bla*_ACT_ *, bla*_FOX_ *, bla*_CMY_ *, bla*_ACC_ *, bla*_MOX_ *, bla*_CIT_ *, bla*_EBC_	0 (0)	0 (0)	0 (0)	0 (0)	0 (0)

### Distribution of hypervirulence-associated genes and hypervirulent strains

Hypervirulence-associated genes have been reported in some clinical isolates of pathogenic bacteria, and they contributed to hypervirulence and resulted in more serious illness (Zhang et al., 2020; Han et al., 2022). In this study, 45 (28.66%) carbapenem-resistant *Klebsiella pneumoniae* (CRKP) strains carried hypervirulence genes, which were *iucA* (24.84%, 39/157), *_p_rmpA* (17.83%, 28/157), *peg-344* (17.20%, 27/157) and *_p_rmpA2* (9.55%, 15/157). The other hypervirulent genes *_c_rmpA*, *ter*B, *iroB* and *irp*2 were not detected. In addition, the hypervirulent genes of *Escherichia coli*, *Stx1, Stx2 and eae*, were not detected in this study (Table 4). Furthermore, 22 CRKP strains with hypervirulence genes had a hypermucoviscous phenotype (a positive string test result) as well, which suggested that carbapenem-resistant and hypervirulent *Klebsiella pneumoniae* (CR-HVKP) were also detected in this study.

**Table 4:** The hypervirulence-associated genes of *Klebsiella pneumoniae* and *Escherichia coli* in this study.

Hypervirulent genes, *n* (%)	*Klebsiella pneumoniae*	Hypervirulent genes, *n* (%)	*Escherichia coli*
	*n* = 157		*n* = 103
*iucA*	39 (24.84)	*Stx1*	0 (0)
*_p_rmpA*	28 (17.83)	*Stx2*	0 (0)
*peg-344*	27 (17.20)	*eae*	0 (0)
*_p_rmpA2*	15 (9.55)		
*_c_rmpA*	0 (0)		
*terB*	0 (0)		
*iroB*	0 (0)		
*irp2*	0 (0)		

### Clinical characteristics of CR-HVKP infection

**Figure 3:**
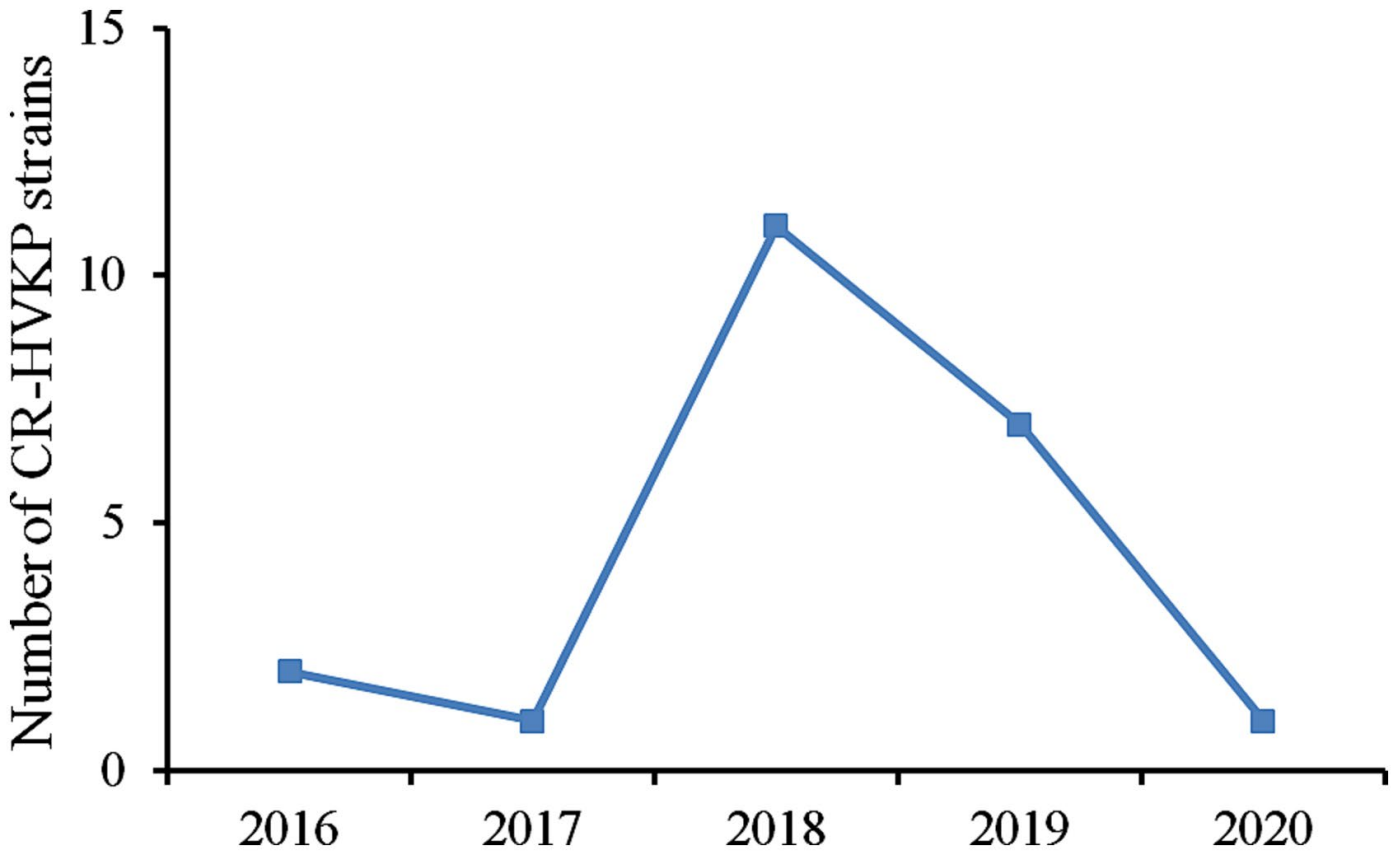
The number of CR-HVKP strains isolated from 2016 to 2020 in this study.

In this study, a small number of CR-HVKP strains were detected in 2016 and 2017 (2/22 and 1/22, respectively). While in 2018, the number of CR-HVKP strains was increased remarkably, accounting for 50% (11/22) strains in this study. However, their number decreased during 2019 and 2020 (7/22 and 1/22, respectively) (Figure 3). The clinical characteristics of the CR-HVKP infection in this study are summarized in Table 5. The percentage of pediatric patients in PICU infected with CR-HVKP was much higher than those in PICU infected with non-hypervirulent CRKP (*p* < 0.05). Besides, more percentage of pediatric patients infected with CR-HVKP suffered from pneumonia than those infected with non-hypervirulent CRKP (*p* < 0.05). Notably, the average hospitalization time of children infected by CR-HVKP strains was longer than that of children infected by non-hypervirulent CRKP, and the antimicrobial therapy was less effective according to physician judgment (*p* < 0.01).

**Table 5:** Clinical characteristics and epidemiology of CR-HVKP strains identified in this study.

	Total CRKP	CR-HVKP	Non-hypervirulent CRKP
	*n* = 157	*n* = 22	*n* = 135
Gender, *n* (%)			
Male	85 (54.14)	12 (54.55)	73 (54.07)
Female	72 (45.86)	10 (45.45)	62 (45.93)
Age			
Median	1 m	2 m	1 m
Range	1 h–16 y	1 d–12 y	1 h–6 y
Age, *n* (%)			
<5 y	145 (92.36)	20 (90.91)	125 (92.59)
<1 m	74 (47.13)	10 (45.45)	64 (47.41)
1–6 m	47 (29.94)	7 (31.82)	40 (29.63)
7–12 m	10 (6.37)	1 (4.55)	9 (6.67)
13–59 m	14 (8.92)	2 (9.09)	12 (8.89)
5–18 y	12 (7.64)	2 (9.09)	10 (7.41)
Specimen			
Sputum	75 (47.77)	10 (45.45)	65 (48.15)
Urine	52 (33.12)	7 (31.82)	45 (33.33)
Blood	15 (9.55)	2 (9.09)	13 (9.63)
Alveolar lavage fluid	5 (3.18)	1 (4.55)	4 (2.96)
Throat swab	4 (2.55)	1 (4.55)	3 (2.22)
Others	6 (3.82)	1 (4.55)	5 (3.70)
Wards, *n* (%)			
Neonatology	46 (29.30)	7 (31.82)	39 (28.89)
NICU	28 (17.83)	3 (13.64)	25 (18.52)
PICU	18 (11.46)	6 (27.27)^a^	12 (8.89)
Hematology	17 (10.83)	2 (9.09)	15 (11.11)
Respiration	10 (6.37)	2 (9.09)	8 (5.93)
Others	38 (24.20)	2 (9.09)	36 (26.67)
Diseases			
Pneumonia	104 (66.24)	19 (86.36)^b^	85 (62.96)
Icterus	42 (26.75)	6 (27.27)	36 (26.67)
Urinary tract infection	39 (24.84)	6 (27.27)	33 (24.44)
Septicemia	32 (20.38)	4 (18.18)	28 (20.74)
Underlying condition			
Cardiac diseases	21 (13.38)	3 (13.64)	18 (13.33)
Leukemia	19 (12.10)	3 (13.64)	16 (11.85)
Urinary disease	7 (4.46)	1 (4.55)	6 (4.44)
Epilepsy	5 (3.18)	0 (0)	5 (3.70)
Respiratory disease	2 (1.27)	1 (4.55)	1 (0.74)
Antibiotics use			
Latamoxef	51 (32.48)	8 (36.36)	43 (31.85)
Cefoperazone/sulbactam	50 (31.85)	7 (31.82)	43 (31.85)
Imipenem	25 (15.92)	4 (18.18)	21 (15.56)
Meropenem	22 (14.01)	3 (13.64)	19 (14.07)
Piperacillin/tazobactam	15 (9.55)	2 (9.09)	13 (9.63)
Others	62 (39.49)	10 (45.45)	52 (38.52)
Hospitalization			
Mean	28 d	33 d	26 d
Range	4 d–116 d	6 d–116 d	4 d–78 d
Antimicrobial therapy outcome			
Effective	120 (76.43)	12 (54.55)^c^	108 (80.00)

a, *p* = 0.03164; b, *p* = 0.03137; c, *p* = 0.00909.

## Discussion

CRE is one of the most common gram-negative bacteria responsible for various nosocomial and community-acquired infections (Perez and Bonomo, 2019; Nordmann et al., 2012; Logan and Weinstein, 2017). It has been increasingly reported globally and the detection rate of CRE in China also increased in recent years (> 20%) (Logan and Weinstein, 2017; Hu et al., 2018). Notably, this rate in children was higher than that in adults (Hu et al., 2018; Wang et al., 2020). In this study, 281 strains of CRE were identified in Children’s Hospital of Soochow University, including CRKP, CREC and other carbapenem-resistant *Enterobacteriaceae*. Fortunately, the detection rate of CRE isolated from pediatric patients in Suzhou was lower than that in other areas of China, and decreased gradually after 2018. In addition, this detection rate in children was also lower than that in adults in China (Zhang et al., 2018). In 2020, its detection rate had nearly halved due to the outbreak of Corona Virus Disease 2019 (COVID-19).

CRE isolates exhibit high resistance to most antimicrobial drugs and limited therapeutic options for clinicians are available (Logan and Weinstein, 2017; Hu et al., 2018). Its drug-resistance mechanism is mainly due to the production of hydrolases, including carbapenemases, ESBLs and AmpC cephalosporinases (Wang et al., 2020). At the present time, *bla*_KPC_, *bla*_NDM_ and *bla*_OXA_ are the most prevalent carbapenemases globally (Miao et al., 2019). In China, *bla*_KPC-2_ is the most common carbapenemase in adults (Wang et al., 2018). However, *bla*_IMP-4_, *bla*_NDM-1_ and *bla*_OXA-232_ were prevalent at different periods in Chinese pediatric patients (Wang et al., 2020; Dong et al., 2018). In this study, most CRE isolates carried carbapenem-resistant genes, including *bla*_OXA-232_, *bla*_KPC-2_, *bla*_OXA-1_, *bla*_NDM-5_ and *bla*_NDM-1_. In addition, most CRE strains harbored the ESBL genes, including *bla*_TEM-1_, *bla*_CTX-M-14_ and *bla*_SHV-11_, and a part of them carried AmpC genes *bla*_DHA-1._ The carriage of these carbapenemase genes explained the high drug-resistance of these CRE strains. However, PCR failed to identify any carbapenemases among 15 out of 281 CRE strains, including 2 *Klebsiella pneumonia*, 2 *Escherichia coli*, 2 *Klebsiella aerogenes*, 2 *Enterobacter cloacae*, 2 *Citrobacter freundii*, 2 *Raoultella ornithinolytica*, 1 *Serratia marcescens*, 1 *Klebsiella oxytoca* and 1 *Salmonella Ughelli*, suggesting that other mechanisms may contribute to the phenotypic carbapenem resistance. Apart from production of carbapenemases, the combination of outer membrane porin dysfunction with hyper-production of AmpC or ESBLs, efflux pumps and penicillin-binding protein modifications may also contribute to carbapenem resistance (Miao et al., 2019). The resistance mechanisms in these non-carbapenemase-producing CRE isolates remain to be determined. These results were consistent with part of previous researches, and diverged from part of other reports, which may be due to the different geographic areas and populations.

Apart from high resistance to antimicrobial drugs, another worrisome event is the increased prevalence of hypervirulent bacteria, like HVKP and HVEC (Russo and Marr, 2019; Divya and Hatha, 2019). Historically, hypervirulent bacteria were susceptible to most antibiotics (Li et al., 2014; Cejas et al., 2014). However, acquisition of a carbapenemase encoding plasmid by hypervirulent *Enterobacteriaceae*, or acquisition of a plasmid with hypervirulence-associated genes by CRE, promote the emergence of carbapenem-resistant and hypervirulent *Enterobacteriaceae*, such as CR-HVKP. These strains often cause severe and life-threatening infections, with a high morbidity and mortality rate (Shon et al., 2013; Ernst et al., 2020). However, limited studies of CR-HVKP isolated from children were reported. In this study, 22 CR-HVKP strains were isolated from pediatric patients. Unfortunately, the infections caused by them were more severe than that caused by non-hypervirulent CRKP. The emergence of CR-HVKP in pediatric patients is challenging for anti-infection treatment and further investigations are required to develop new strategies.

In this study, we investigated the antimicrobial resistance, hypervirulence and clinical characteristics of CRE isolated from children. However, we did not analyse the multilocus sequence typing and whole-genome data of these strains, which may help to elucidate the transmission and evolution of drug resistance and hypervirulence associated genes. Further studies need to address this limitations.

## Data Availability

The raw data supporting the conclusions of this article will be made available by the authors, without undue reservation.
